# Height‐related scaling of phloem anatomy and the evolution of sieve element end wall types in woody plants

**DOI:** 10.1111/nph.14360

**Published:** 2016-12-09

**Authors:** Johannes Liesche, Marcelo R. Pace, Qiyu Xu, Yongqing Li, Shaolin Chen

**Affiliations:** ^1^ College of Life SciencesNorthwest A&F UniversityYangling712100China; ^2^ Biomass Energy Center for Arid and Semi‐arid landsNorthwest A&F UniversityYangling712100China; ^3^ Department of BotanyNational Museum of Natural HistorySmithsonian InstitutionWashingtonDC20013‐7012USA; ^4^ South China Botanical GardenChinese Academy of SciencesGuangzhou510650China

**Keywords:** correlation analysis, evolution, phloem, phylogeny, sieve element, sieve plate, sieve pore, trees

## Abstract

- In the sieve elements (SEs) of the phloem, carbohydrates are transported throughout the whole plant from their site of production to sites of consumption or storage. SE structure, especially of the pore‐rich end walls, has a direct effect on translocation efficiency. Differences in pore size and other features were interpreted as an evolutionary trend towards reduced hydraulic resistance. However, this has never been confirmed.
- Anatomical data of 447 species of woody angiosperms and gymnosperms were used for a phylogenetic analysis of end wall types, calculation of hydraulic resistance and correlation analysis with morphological and physiological variables. end wall types were defined according to pore arrangement: either grouped into a single area (simple) or into multiple areas along the end wall (compound).
- Convergent evolution of end wall types was demonstrated in woody angiosperms. In addition, an optimization of end wall resistance with plant height was discovered, but found to be independent of end wall type. While physiological factors also showed no correlation with end wall types, the number of sieve areas per end wall was found to scale with SE length.
- The results exclude the minimization of hydraulic resistance as evolutionary driver of different end wall types, contradicting this long‐standing assumption. Instead, end wall type might depend on SE length.

## Introduction

Carbohydrates are the primary units of carbon and energy transported inside plants. From leaves, where they are produced via photosynthesis, they are distributed to other organs according to demand for growth or storage. The transport of carbohydrates requires a highly efficient pathway, especially in trees, where distances between carbon source and sink organs reach tens of meters (Ryan & Asao, 2014). A continuous, low‐resistance pathway is provided by the conducting cells of the phloem, the sieve elements (SEs). These cells are elongated in shape and, in the mature state, devoid of most cytoplasmic content. Importantly, SEs are axially connected via open pores, in the case of angiosperms, or via numerous plasmodesmata‐like cell wall channels in the case of gymnosperms. Theoretical models state that the SE end walls, also called sieve plates in angiosperms, are a decisive factor in determining the conductivity of the transport path (Thompson & Holbrook, 2003).

**Figure 1:**
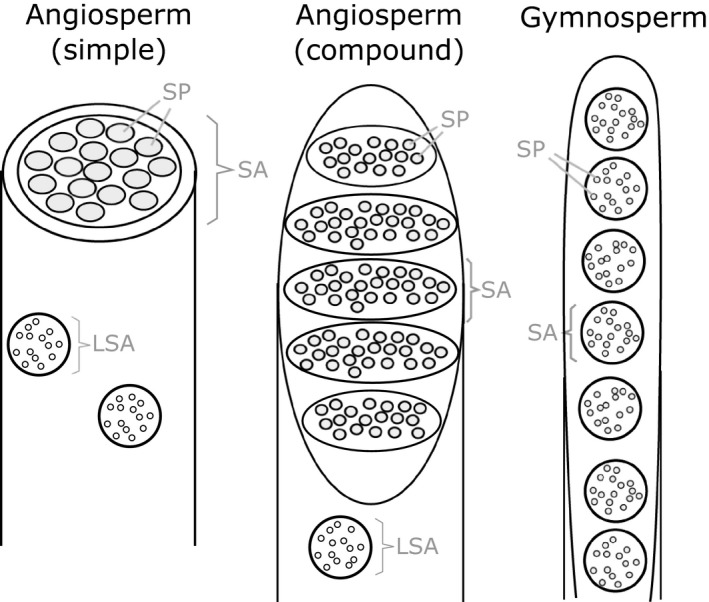
Schematic drawing of phloem sieve element end wall types. Some angiosperm species have a transverse‐oriented end wall covered by a single sieve area (SA) (simple), while others have inclined end walls with, typically, between two and 20 sieve areas (compound). Gymnosperms have highly inclined end walls with, typically, between 12 and 30 sieve areas. While in angiosperms sieve areas on the end walls are different from lateral sieve areas (LSA), there is no distinct difference between sieve areas in gymnosperms. SP, sieve pore.

Comparative studies of SE end wall anatomy in a wide range of species have been conducted and, based on their results, different end wall types have been defined (Fig. 1; MacDaniels, 1918; Esau & Cheadle, 1959; Zahur, 1959). One type refers to transverse‐oriented end walls that are covered by a single sieve area. Another type refers to compound end walls, which typically have inclined orientation and contain multiple sieve areas, which can be scalariform or reticulate. Often, an additional type is defined for species that show both simple and compound end walls (Esau & Cheadle, 1959; Angyalossy *et al*., 2016). Gymnosperms have end walls that reunite numerous sieve areas but without forming sieve plates, as pores at the end wall are equal in width to those of the lateral walls. In this sense, gymnosperms differ from angiosperms, which have specialized sieve areas with wider sieve pores reunited in the end walls, known as the sieve plates.

Indeed, the characteristics of end wall anatomy that were described after comparative analyses of large numbers of species (Fig. 1) were interpreted as an evolutionary trend towards higher conductivity (Esau & Cheadle, 1959; Zahur, 1959; Esau, 1969; Den Outer, 1983). However, so far, no evidence has been produced to confirm that the minimization of end wall resistance is the evolutionary driver for end wall anatomy. By contrast, a recent investigation of phloem diversity and evolution in the tribe Bignonieae (Bignoniaceae) showed that phloem features, especially end wall anatomy, show great intrafamily variability that cannot be explained by a single evolutionary trend, such as towards optimization of resistance (Pace *et al*., 2015). Furthermore, experiments performed on several species of herbaceous plants did not show a correlation between end wall conductivity and phloem transport efficiency (Mullendore *et al*., 2010).

The aims of this study were to re‐evaluate and combine data for a more complete phylogeny of end wall types, and to explore correlations of end wall anatomy with morphological and physiological factors in order to elucidate the evolutionary pressure that led to their specific development in the various species.

One critical aspect of this analysis is the measurement of sieve pore radii, which has been the subject of discussions in the past. On fixed samples, the pores often appear partly occluded by callose depositions, which some authors assumed to be present in the physiological, conductive state, as callose platelets participate in the formation of sieve pores (Esau & Cheadle, 1959). However, subtler fixation techniques and live cell imaging showed that callose deposition is mainly a wound reaction (Eschrich, 1975; Evert, 1990; Schulz, 1992). Therefore, sieve pore radii were determined here, including the callose ring, if present, providing values for the maximal conductive area. In a conducting SE, this maximal pore area can be reduced by P‐protein filaments in angiosperms (Cronshaw & Sabnis, 1990) and endoplasmic reticulum membrane aggregates in gymnosperms (Schulz, 1992), but the influence of these pore contents can be assumed to be similar in all species of the respective groups.

This study concentrates on woody plants, because their extensive secondary growth constitutes a principal difference from herbaceous plants. The capability of generating new phloem, instead of relying on a limited number of cells, enables woody plants to increase transport capacity when needed. This flexibility might entail a different application of evolutionary pressures compared with herbaceous plants. We also considered some arboreal monocotyledons, such as palms. Although typically lacking secondary growth, the SEs of palms differ from those of herbaceous plants for remaining conductive for the entire palm life span, which can be several decades (Tomlinson *et al*., 2011).

## Materials and Methods

### Sampling and microscopic analysis

All samples were obtained from fresh material, collected either from natural plant populations or from cultivations (botanical gardens, arboretums) within each plant's natural range. Only mature trees that reached the typical maximum height were sampled. The procedure for sampling and analysis resembles the procedure followed in previous investigations of SE end wall anatomy (MacDaniels, 1918; Esau & Cheadle, 1959; Pace *et al*., 2015). A 1 × 2 cm piece of bark was cut from the stem at breast height (1.3 m) using a sharp knife, immediately fixed in 70% formalin‐acetic acid alcohol (Berlyn & Miksche, 1976) and subsequently stored in 70% ethanol. Samples were embedded in historesin (Leica Microsystems, Mannheim, Germany). To perform historesin embedding, 2 mm cubes containing xylem, cambium and contiguous phloem were placed for *c*. 1 month in pre‐embedding solution and then 1 month in embedding solution within a refrigerated vacuum chamber. Afterwards, the samples were placed in molds, later sectioned with the aid of a rotary microtome and, finally, stained in 0.05% toluidine blue O in glacial acetic buffer at pH 4.7 (O'Brien *et al*., 1964). Sections of these materials were made with the aid of a sliding microtome (Leica Biosystems, Wetzlar, Germany), using a foam resin to prevent the samples from tearing apart and preserving, as well as possible, the fragile phloem, following the technique proposed by Barbosa *et al*. (2010). Images of samples were obtained by bright‐field light microscopy (Leica Microsystems). In addition to fixed material, some samples of trees with simple end walls were analyzed without fixation. Instead, hand sections of these samples were produced immediately after sampling, stained with 0.05 mg ml^−1^ Aniline Blue (Sigma Aldrich) for 5–10 min and analyzed by fluorescence and bright‐field microscopy using an Olympus Fluoview microscope (Olympus, Tokyo, Japan) equipped with a ×100 objective (numerical aperture 1.3) and digital camera DP10 (Olympus).

On all images, SE diameter, length, end wall thickness, number of pores and pore diameters were determined in imagej (Schindelein *et al*., 2012). Only images that show complete sieve area were used for the measurements of pore diameters. As the sieve pores of all the species sampled here were round, pore diameter could be determined by drawing a measurement line at random orientation. The pore diameter was measured including the callose collar, which is the result of experimental procedures and is not expected to be present in the cells' functional state (Mullendore *et al*., 2010). The secondary phloem can be divided into conducting and nonconducting phloem (Angyalossy *et al*., 2016). All comparisons and measurements were carried out exclusively in the conducting phloem, except for qualitative descriptions of sclerenchyma, which involved the nonconducting phloem. In addition to the microscopic analysis, stem length and leaf length were measured. Stem length was determined using a photograph of the tree together with a height reference. Leaf length was measured as the average of 10 leaves from the middle to lower crown area. In total, data from 89 species was obtained experimentally.

### Compilation of data from literature sources

In order to create a comprehensive data set, the experimental data for 89 species were combined with values found in the literature for 474 species, resulting, after subtraction of overlap, in a total number of species of 447. The complete data including references are given in a supplementary spreadsheet (Supporting Information Table S1). Most of the literature data on SE and end wall characteristics stem from MacDaniels (1918), Chang (1954) and Zahur (1959), which, in total, provided values for 308 species of woody angiosperms and gymnosperms. As image material is only given for a fraction of these species, we validated their accuracy by our own measurement for 27 species, randomly chosen from available plants. The critical value of sieve pore radius given by MacDaniels (1918) and Chang (1954) was found to be in good agreement with our own data, confirming that the authors included the callose collar in their measurements. Data from additional studies were used only when they included images that could be used to confirm measurements.

As the data on phloem anatomy were usually not accompanied by values for the other parameters used in this study, these were collected from other sources. Values of average stem length, leaf length of mature plants and their native climate zone were obtained from floras (eFloras, 2008). Growth rates were obtained from Dr Nathaniel Stephenson (US Geological Survey). The key parameters of number of sieve areas per end wall and stem length were determined for all species. However, not all of the other parameters could be determined for all species, and the availability of specific parameters is indicated in the results section and Table S1.

### Phylogeny

The phylogenetic trees were assembled in Mesquite 3.04 (Maddison & Maddison, 2015), based on information from the angiosperm phylogeny website (Stevens, 2001 onwards) and recent publications on the phylogeny of specific families, as detailed in Fig. S1. End wall character was mapped on the trees using parsimony reconstruction of ancestral states. Species were classified according to their average number of sieve areas per end wall. Species with > 50% end walls with multiple sieve areas were classified as compound end walls, while others were classified as having simple end walls. The group of plants recorded as having both simple and compound end walls was relatively small. Only 6% of species had an average number of sieve areas per end wall > 1, but < 2.5.

### Statistics

The values for anatomical parameters constitute averages derived from the analysis of at least 25 SEs and end walls per sample. While values taken from literature sources are often based on the analysis of > 20 SEs (Zahur, 1959; Den Outer, 1983), some sources did not specify how many SEs or end walls were analyzed. Data from these sources constitute 7% of the total data. Samples were, in most cases, obtained from a single tree of each species. An earlier investigation that sampled different individuals from 33 species in various habitats established that there is no difference in end wall characteristics of different trees of the same species at the same developmental stage (MacDaniels, 1918). This conclusion was supported here by measurement of multiple individuals of 16 species, which did not show significant differences, as tested with Student's *t*‐test, in the number of sieve areas, sieve pore radius, SE radius and length (Table S2).

Correlation between two parameters was tested using the Pearson product moment correlation method. This allows the strength of the association between pairs of variables to be measured, and it also allows us to determine whether the relationship, if any, between the variables is a straight line. It is a parametric test that assumes the residuals are normally distributed with constant variance. These criteria were tested for using the Shapiro–Wilk test and Spearman rank correlation. Both criteria were found to be satisfied in parameter pairs that showed significant correlation. Only the pairs of variables with *P*‐value < 0.05 were considered significantly correlated. Furthermore, the correlation is described as strong for pairs with a Pearson product moment correlation coefficient > 0.5 and weak for pairs with a coefficient between 0.3 and 0.5. If these statistical tests indicated a correlation, a regression line was added to the respective graphs using the least‐square estimator function. All calculations were performed in sigmaplot 12.5 (Systat Software, San Jose, CA, USA).

### Calculation of hydraulic resistance

The hydraulic resistance of end walls *R*_EW_ was calculated as described by Jensen *et al*. (2012b) and Liesche *et al*. (2015) with

(Eqn 1) $$R_{\mathrm{EW}}=\frac{1}{N}\left(\frac{3\eta }{r_{p}^{3}}A+\frac{8\eta l_{p}}{\pi r_{p}^{4}}B\right).$$

It includes variables for the number of pores *N*, the phloem sap viscosity *η*, the pore radius *r*_p_ and the pore length *L*, which was measured here as the end wall thickness. The first term in the bracket describes the viscous friction at the pore entrance and the second term is the Hagen–Poiseuille factor accounting for the pore lumen resistance. In addition, *A* and *B* are factors that account for the influence of variability in pore radii on resistance, including the SD, *σ*, and are given by

$$A=\frac{1}{1+3{\left(\frac{\sigma }{r_{p}}\right)}^{2}},B=\frac{1}{1+6{\left(\frac{\sigma }{r_{p}}\right)}^{2}+6{\left(\frac{\sigma }{r_{p}}\right)}^{4}}.$$

The hydraulic resistance of SE lumen *R*_L_ was calculated as described by Jensen *et al*. (2012b) with

(Eqn 2) $$R_{L}=\frac{8\eta l}{\pi r^{4}},$$

using variables for SE length *l* and SE radius *r*. A value of *η* = 2 mPa s was assumed for phloem sap viscosity in all species, in line with previous theoretical descriptions of phloem flow (Thompson & Holbrook, 2003; Mullendore *et al*., 2010).

## Results

### The variety of end wall anatomies encountered in many families contradicts an evolutionary trend towards a specific end wall type

**Figure 2:**
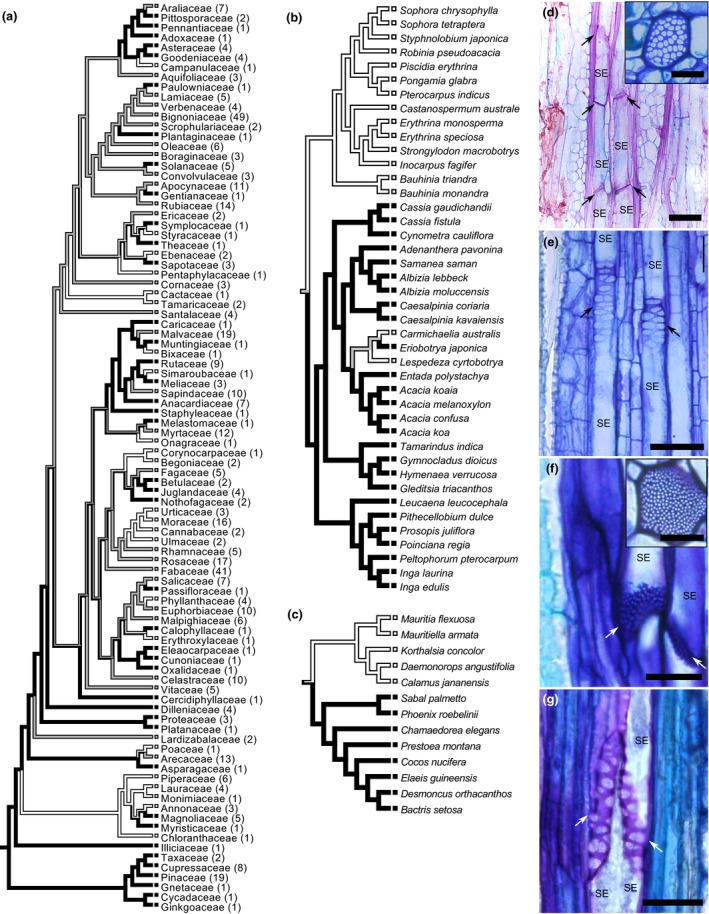
Phylogenetic distribution of the simple and compound types of sieve element end walls. (a) Phylogenetic tree with sieve element end wall types mapped. The number of species representing each family is provided in brackets. (b–g) Detailed phylogeny of Fabaceae (b) and Arecaceae (c) and exemplary images of representatives with simple (d, f) and compound (e, g) end walls: (d) *Strongylodon macrobotrys*; (e) *Entada polystachya*; (f) *Mauritiella armata*; (g) *Bactris setosa*. See Supporting Information Fig. S1 for phylogenetic trees of all families with variations in end wall type. White bars, simple end walls; black bars, compound end walls; gray bars, occurrence of both types. Arrows in (d–g) highlight sieve element end walls. SE, sieve element. Bars, 20 μm (insets 10 μm).

The occurrence of different end wall types has generally been discussed in terms of evolutionary specialization, even though the identification of families with representatives of simple and compound end walls suggested flexibility in the evolution of this trait (MacDaniels, 1918; Donghua & Xinzeng, 1993; Wenqing & Xinzeng, 1994; Pace *et al*., 2015). The data for 447 species of woody plants from 93 families obtained from previously published studies and our own measurements provide an overview of the phylogenetic distribution of end wall types (Fig. 2).

The phylogenetic tree clearly shows the convergent evolution to both simple and compound end wall types (Fig. 2). Simple end walls developed from ancestors with compound end walls in numerous species independently. Examples are *Fraxinus nigra* and *excelsior* or *Fagus grandifolia* and *sylvatica* (Fig. S1). Similarly, compound end walls occur in species whose ancestors probably had simple end walls, for example *Coprosma grandifolia* or *Tamarindus indica*. Variation of end wall type was found in 25 of the 46 families that are represented by at least three species (Fig. 2a). While the distribution in some families matches the species' association with different tribes, variation within tribes or within genus is also present (Figs 2b,c, S1).

### End wall anatomy is linked to stem length

**Figure 3:**
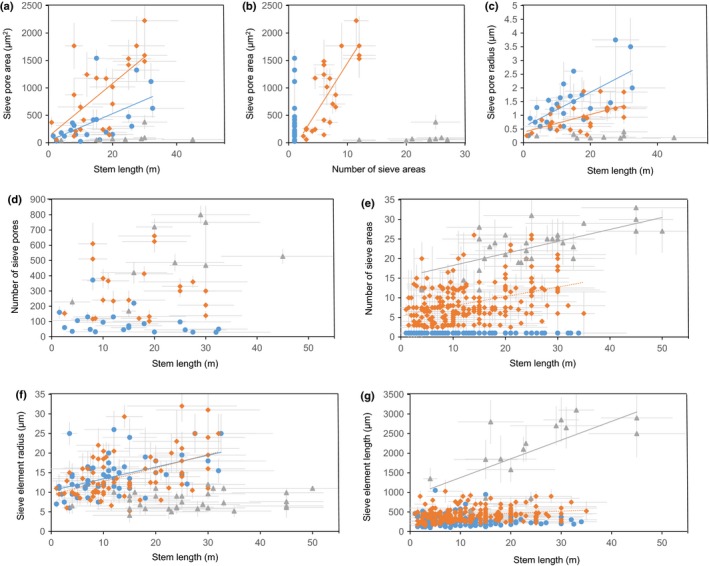
Relationship of stem length to anatomical parameters of sieve elements and their end walls in woody angiosperms with simple end walls (blue circle), woody angiosperms with compound end walls (orange diamond) and woody gymnosperms (grey triangle). (a) Relationship between stem length and total sieve pore area per end wall. (b) Relationship between sieve pore area and number of sieve areas per end wall. (c–g) Relationship between stem length and average sieve pore radius (c), number of pores per end wall (d), number of sieve areas (e), sieve element radius (f) and sieve element length (g). Regression lines were only added in case there was a significant correlation between two parameters. Solid line, strong correlation; dotted line, weak correlation.

The extensive data allowed us to search for correlations of end wall type with physiological parameters. A correlation would indicate that the parameter was a potential driver for the development of a certain end wall type in a given species. Earlier analyses have focused solely on the relationship of end wall type to other characteristics of the phloem and/or bark tissues (Zahur, 1959; Den Outer, 1983; Chavan, 1986; Pace *et al*., 2015). Mathematical modeling based on phloem anatomy suggested that trees use the same mechanism of osmotically driven pressure flow for phloem transport as herbaceous plants (Jensen *et al*., 2012a). This means that transport speed depends on a single source–sink hydrostatic pressure gradient, which, in trees, must reach over distances of tens of meters. In accordance with results from the vine *Ipomoea nil* (Knoblauch *et al*., 2016), we hypothesized that increased stem length is accompanied by the optimization of end wall and SE anatomy for conductivity. Results from the correlation analysis of stem length and anatomical parameters that influence phloem conductivity are presented later (Fig. 3; Table 1). The hydraulic resistances that were calculated based on these parameters according to a current model are provided in the following section.

**Table 1:** Results of Pearson product moment correlation and linear regression analysis

Parameter 1	Parameter 2	Group	Pearson product moment correlation coefficient	Probability level	Number of samples	*r* ^2^	Standard error of regression	Mathematical relationship
Sieve pore area	Stem length	Angiosperms (simple)	0.559	0.00839	21	0.313	361.14	31.08 + (24.96 × *X*)
		Angiosperms (compound)	0.655	0.0007	23	0.428	502.63	113.29 + (48.29 × *X*)
		Gymnosperms	0.272	0.479	9			No significant correlation
Sieve pore area	Number of sieve areas	Angiosperms (compound)	0.778	0.000012	23	0.606	417.53	−244.69 + (169.81 × *X*)
		Gymnosperms	0.325	0.394	9			No significant correlation
Sieve pore radius	Stem length	Angiosperms (simple)	0.672	0.00023	25	0.452	0.64	0.57 + (0.063 × *X*)
		Angiosperms (compound)	0.577	0.00164	27	0.333	0.397	0.38 + (0.032 × *X*)
		Gymnosperms	0.598	0.089	9			No significant correlation
Number of pores per end wall	Stem length	Angiosperms (simple)	−0.318	0.16	21			No significant correlation
		Angiosperms (compound)	−0.0154	0.946	22			No significant correlation
		Gymnosperms	0.558	0.119	9			No significant correlation
Number of sieve areas	Stem length	Angiosperms (compound)	0.388	5.10E‐11	239	0.147	7.16	6.99 + (0.627 × *X*)
		Gymnosperms	0.671	2.60E‐05	32	0.432	8.34	−7.9 + (1.472 × *X*)
Sieve element radius	Stem length	Angiosperms (simple)	0.504	5.00E‐05	59	0.254	4.2	10.42 + (0.301 × *X*)
		Angiosperms (compound)	0.493	7.00E‐06	75	0.233	7.33	4.06 + (0.71 × *X*)
		Gymnosperms	0.158	0.387	32			No significant correlation
Sieve element length	Stem length	Angiosperms (simple)	−0.026	0.754	145			No significant correlation
		Angiosperms (compound)	0.344	3.00E‐04	215	0.129	155.5	363.4 + (5.943 × *X*)
		Gymnosperms	0.657	0.0057	16	0.432	639	899.6 + (47.84 × *X*)
Number of sieve areas	Sieve element length	All angiosperms	0.597	3.10E‐35	351	0.356	3.93	−1.13 + (0.0173 × *X*)

Pairs of variables with a correlation coefficient > 0.3 and a *P*‐value < 0.05 were considered significantly correlated.

The analysis of total sieve pore area, that is, the sum of the areas of all sieve pores per end wall, revealed a positive correlation of sieve pore area to stem length in angiosperms, but the same was not true for gymnosperms (Fig. 3a). The increase is slightly steeper for angiosperms with compound end walls compared with species with simple end walls (Fig. 3a). A closer look at the relation of sieve pore area per end wall does, furthermore, show that it increases with the number of sieve areas in angiosperms that have compound end walls (Fig. 3b). As the sieve pore area shows limited variation in gymnosperms, there is no correlation to the number of sieve areas per end wall (Fig. 3b). For angiosperms with simple or compound end walls, the higher sieve pore area with higher stem length is the result of an increased average pore radius (Fig. 3c), rather than an increase in the number of pores (Fig. 3d). In gymnosperms, there is no significant correlation of stem length with the number of pores (Fig. 3d) or with the sieve pore radius (Fig. 3c), which results in the constancy of sieve pore area with stem length (Fig. 3a). The number of sieve areas shows a strong correlation with stem length in gymnosperms (Fig. 3e).

Changes in end wall anatomy with stem length are accompanied by changes in SE length and radius, the other two major factors that determine the total SE resistance. SE radius shows a strong correlation with stem length in angiosperms with simple end walls, a weak correlation in angiosperms with compound end walls and no correlation in gymnosperms (Fig. 3f). Longer SEs lead to a reduction of hydraulic resistance per unit length because of the lower number of end walls along the phloem path. A strong correlation of stem length with SE length is apparent in gymnosperms, while the parameters show a weak correlation in angiosperms with compound end walls (Fig. 3g).

### Simple and compound end walls can cause similar hydraulic resistance

In order to answer the question of whether stem length‐related changes to SE and end wall anatomy are the driver for evolution towards a specific end wall type, we calculated hydraulic resistances based on the anatomical data presented earlier. As one large pore has a higher conductivity compared with several small pores of the same total cross‐sectional area, the combined pore area per end wall as presented earlier can only serve as a rough estimate of resistance. Calculations were performed according to the prevalent model for the hydraulic resistance, which takes the variability of sieve pore radii on a given end wall into account (Eqn 1; Jensen *et al*., 2012b; Liesche *et al*., 2015).

**Figure 4:**
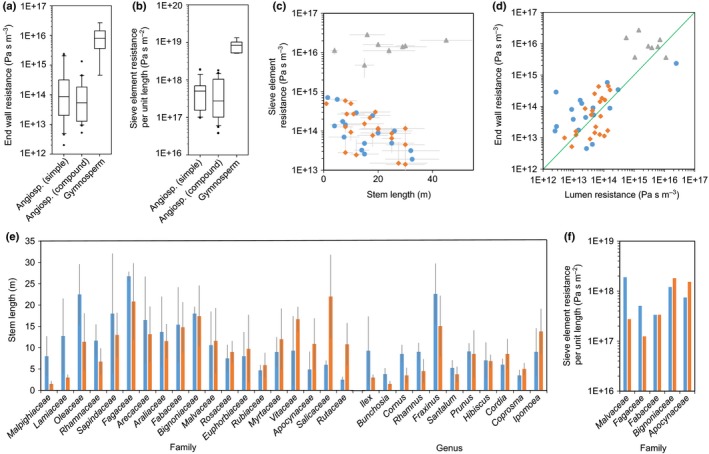
Relationship between sieve element end wall type and hydraulic resistance. (a, b) Box plots of end wall resistance calculated according to Eqn 1 (a), and sieve element resistance per unit length calculated according to Eqn 2 (b) show similar average values for angiosperms species with simple end walls and angiosperms with compound end walls, but higher values for gymnosperms. (c) Differences between the angiosperms and gymnosperms (gray triangles) are visible for sieve element resistance per unit length over the whole range of stem lengths, but not between angiosperms with simple (blue circles) and compound end walls (orange diamonds). (d) The relationship of sieve element lumen to end wall resistance was found to be close to a 1 : 1 ratio (green line) for all plants. (e, f) Closely related angiosperm species with different end wall type show no relationship between end wall type and stem length (e) or sieve element resistance per unit length (f). Blue bars, simple end walls; orange bars, compound end walls. Error bars indicate ± SD.

The average end wall resistance of angiosperms with compound end walls is lower than that of species with simple end walls, although the difference is not significant (Fig. 4a). The average end wall resistance of gymnosperms is about two orders of magnitude larger than that of angiosperms (Fig. 4a). The average values for total SE resistance per unit length for angiosperms with compound and with simple end walls are not significantly different (Fig. 4b), despite the lower number of end walls along the stem of angiosperms with compound end walls (Fig. 3g). This is because of the higher SE radius found in angiosperms with simple end walls (Fig. 3f). The much longer SEs in gymnosperms (Fig. 3g) contribute to a total SE resistance per unit length that is *c*. 10 times higher than that of angiosperms, despite the end wall resistance being *c*. 100 times higher (Fig. 4b) and the narrower SEs (Fig. 3f). The similar average values for the two end wall types in angiosperms are not caused by overrepresentation of specific stem lengths, but reflect the minor differences in resistance at all stem lengths (Fig. 4c). The ratio of SE lumen to end wall resistance was close to 1 : 1 for all species, independent of end wall type (Fig. 4d). This is in accordance with a previous description of the universality of this relationship in herbaceous angiosperms (Jensen *et al*., 2012b) and a limited number of woody angiosperms and gymnosperms (Liesche *et al*., 2015).

These data suggest that both end wall types can result in similar hydraulic resistance in angiosperms. Gymnosperms show an approx. 10 times higher resistance, in line with earlier reports that only included a small sample number (Liesche *et al*., 2015). Direct comparison of average stem length of species with different end wall types of the same family or the same genus confirmed that there is no bias towards higher or lower stem length (Fig. 4e) or higher or lower total SE resistance per unit length (Fig. 4f) for the two end wall types in woody angiosperms.

### End wall types and the mechanical property of the bark

Different end wall types correlate with different mechanical tissue types as described by Den Outer (1983) and Zahur (1959). The mechanical tissue types found in the bark are fibers, which are derived from the cambium and frequently appear in tangential bands, and sclereids, which are derived from belated sclerosis of parenchyma cells in the nonconducting phloem and typically appear in clusters (Esau, 1969; Angyalossy *et al*., 2016). In addition, fiber‐sclereids are, like sclereids, derived from parenchymatous cells, but resemble the shape and growth pattern of fibers (Parameswaran, 1980). The earlier analyses found that most tree species with more than five sieve areas per end wall have fibers arranged in continuous bands, while most tree species with simple end walls have scattered sclereids or no mechanical tissues at all (Den Outer, 1983). In Bignoniaceae, collapse of the SEs has never been recorded in the phloem of fibrous species, whose SEs bear compound end walls, not even in the nonconducting phloem. By contrast, of the species featuring scarce fibers, whose SEs have simple or compound end walls with few sieve areas, most experience total collapse of their SEs in the nonconducting phloem (Pace *et al*., 2015). This shows that the mechanical tissue type can be of high significance for phloem physiology and a potential evolutionary driver towards multiseason usage of SEs.

**Figure 5:**
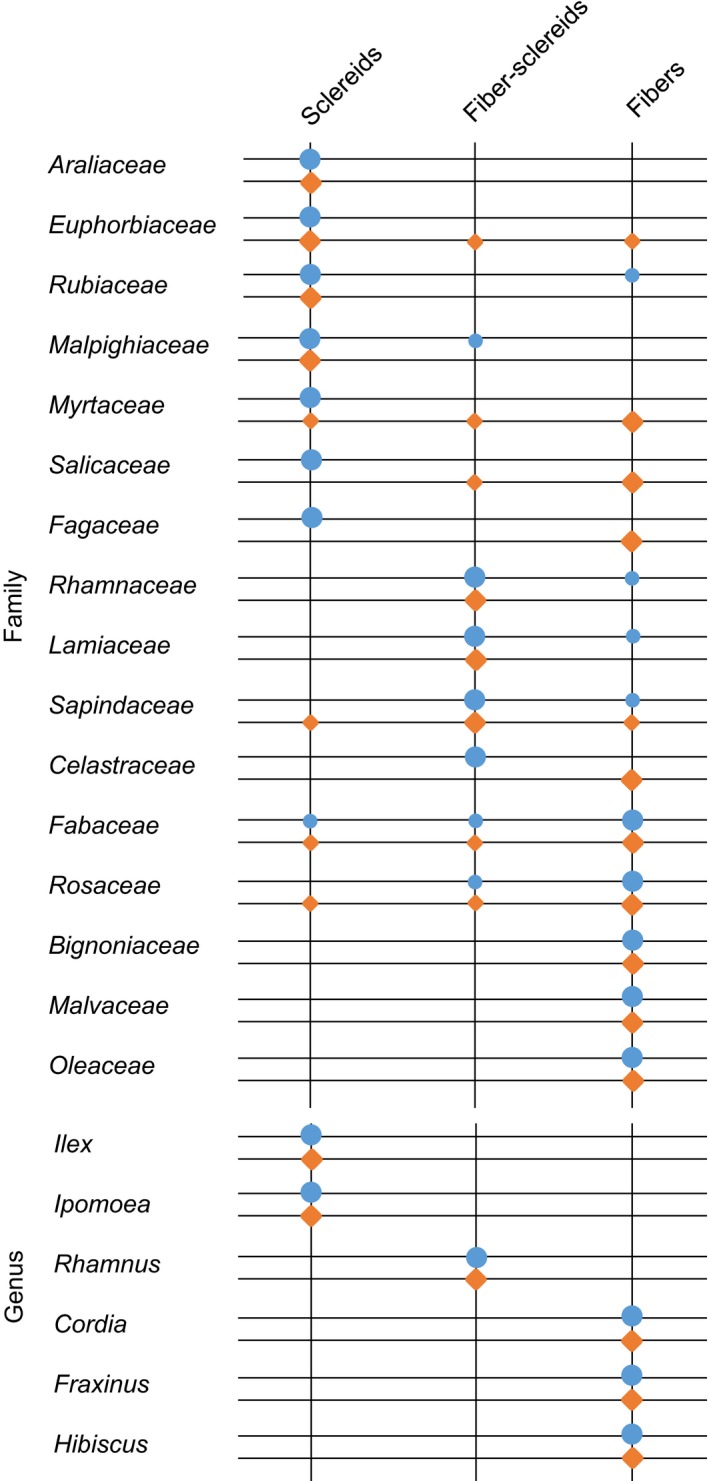
Mechanical tissue types found in families and genera that contain species with different sieve element end wall types. The most abundant type is marked by large circles, while other types also represented are marked by small circles. Blue circles, simple end walls; orange diamonds, compound end walls.

In the analysis of 154 species within 16 families, representatives of which have different end wall types, no correlation with mechanical tissue type was apparent. The most abundant mechanical tissue type within a family was, in 12 out of 16 cases, the same for species with simple and compound end walls (Fig. 5). In several families, a large variety of mechanical tissue types could be found, but this was generally not correlated with end wall type. In the Fabaceae, for example, all mechanical tissue types are represented, both in the group of species with simple end walls and in the group of species with compound end walls. At the genus level, the mechanical tissue type was the same for species with different end wall types in all cases (Fig. 5), contradicting the notion of coevolution of mechanical tissue type and SE end wall type.

### Different end wall anatomies are not related to a plant's native climate zone, phloem loading capacity or growth rate

**Figure 6:**
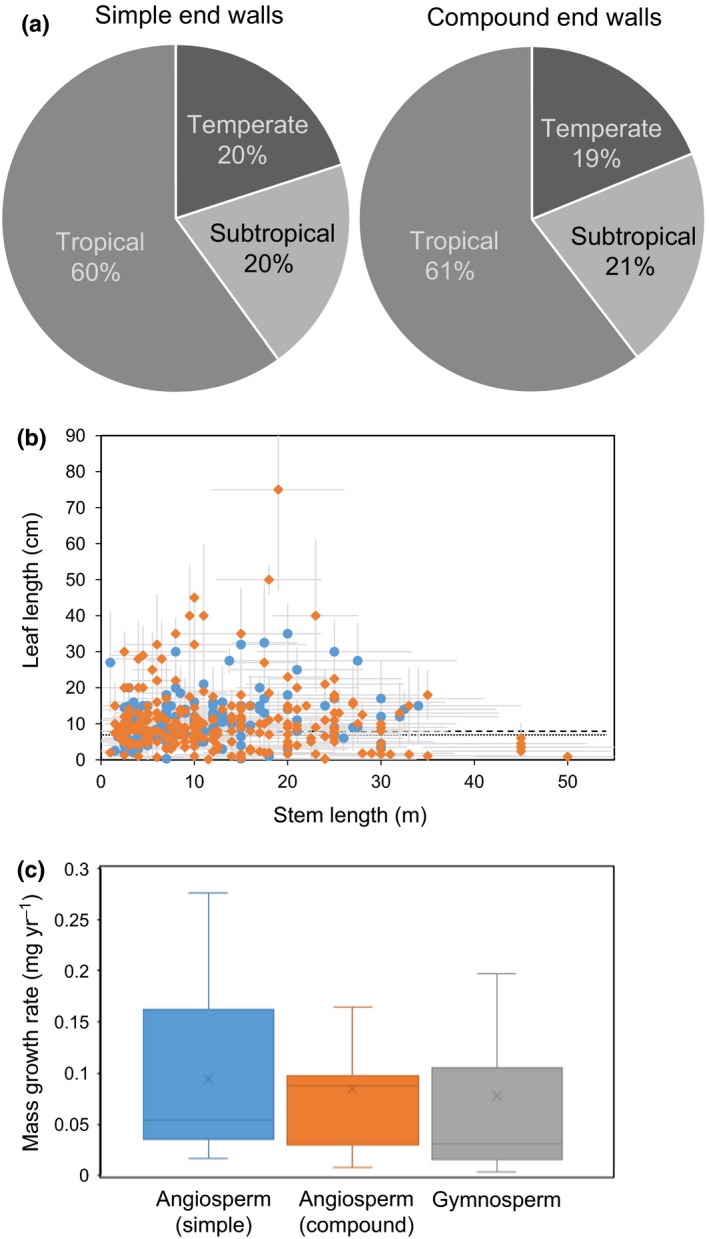
Relationship of sieve element end wall type and native climate zone, leaf length and growth rate. (a) Relationship between native climate zone and end wall type of woody angiosperms, 161 with simple and 242 with compound end walls. (b) Relationship between stem length and leaf length as an indicator of source capacity for woody angiosperms with simple end walls (blue circles) and compound end walls (orange diamonds). Average values are indicated by a dotted line for angiosperms with compound end walls and by a broken line for angiosperms with simple end walls. (c) Box plot of growth rates (annual mass increase) of mature trees of groups with different end wall types. Error bars indicate ± SD.

As stem length and hydraulic resistance were excluded as the drivers for development of a specific end wall type in woody angiosperms, additional physiological parameters were tested. A potential connection of phloem anatomy and a plant's native climate zone was generally not supported. Of 403 woody angiosperms, 161 with simple end walls and 242 with compound end walls, representatives of tropical, subtropical and temperate climate zones were found with similar abundance (Fig. 6a). Furthermore, there was no correlation between end wall type and climate zone within families with many representatives from different climate zones, such as *Fabaceae*,* Moraceae* or *Rosaceae* (Table S1).

An important factor for phloem transport is the osmotic potential that causes the hydrostatic pressure difference between carbohydrate source and sink, the driving force for mass flow of phloem sap. Currently, it is not possible to obtain reliable values for the osmotic pressure potential inside the phloem owing to the SE's inaccessibility and sensitivity to manipulation (Turgeon & Wolf, 2013). Leaf length has been used before as a proxy parameter for phloem loading capacity that showed good agreement with theoretical models (Jensen *et al*., 2012a; Jensen & Zwieniecki, 2013). The average leaf length of angiosperm species in our data set is not significantly different for species with simple end walls (11.5 cm) and those with compound end walls (11.9 cm; Fig. 6b). The average factors are not biased by a specific stem length–leaf length relationship (Fig. 6b).

In addition to carbohydrate import into the phloem, export from the phloem and utilization in the sink tissues influence transport speed and volume. Sink demand of carbohydrates is reflected in the tree's growth rate. The annual stem diameter increase of 42 species of trees of > 10 yr of age was found to be not significantly different for angiosperms with simple end walls, angiosperms with compound end walls and gymnosperms (Fig. 6c).

## Discussion

### Optimization of end wall resistance is linked to stem length

Current theoretical models of phloem transport showed that tall trees need higher hydraulic conductivity in order to match the experimentally observed transport speeds (Mencuccini & Hölttä, 2010) and experimental evidence was recently provided for the vine *Ipomoea nil* (Knoblauch *et al*., 2016). The anatomical data for 447 species of woody angiosperms and gymnosperms revealed a correlation of several resistance‐related parameters with stem length. This suggests that the end wall anatomy of woody plants is modulated in order to allow for lower resistance phloem transport to counteract the negative impact of plant height. This hypothesis is supported by the results of mathematical modeling of SE resistance. Species with simple and compound end walls might represent contrasting strategies to reduce phloem hydraulic resistance. In woody angiosperms with compound end walls, increasing stem length was found to be accompanied by increasing number of sieve areas per end wall, longer and wider SEs and larger sieve pores. In woody plants with simple end walls, stem length was strongly correlated with sieve pore area and SE radius, but not SE length. These anatomical adjustments translate to a reduced total SE resistance, the combination of lumen and end wall resistance, for higher trees. In gymnosperms, stem length was correlated with the number of sieve areas and SE length, but, perhaps because of the limited amount of data, a reduction of SE resistance with stem length was not observed.

The presented results on hydraulic resistance provide an insight into the efficiency of phloem transport in different species and should help to improve current mathematical models of phloem transport that generally neglect this aspect. They support the previously postulated notion of universal optimization of the carbohydrate transport system in all plants (Jensen *et al*., 2012a; Drobnitch *et al*., 2015). However, the results do not relate to actual phloem transport rates, which would require the determination of transport capacity. An analysis of transport capacity, measured as cross‐sectional area of conducting SEs, could show whether trends observed here, for example the scaling of SE diameter with plant height in plants with simple end walls, translate to differences in phloem transport.

### There is no evolutionary trend towards a specific end wall type

While SE resistance is *c*. 10 times higher in gymnosperm trees compared with angiosperm trees, as indicated by previous studies with lower sample number (Liesche *et al*., 2015), there is no significant difference in SE resistance per unit length between woody angiosperm with simple or compound end walls. This result refutes the hypothesis that simple sieve plates are more efficient in conduction compared with compound end walls. This assumption was originally based on data in which the callose collar of sieve pores was considered to be present in the SE's functional state. Accordingly, measurements were made for the inner, callose‐free part of the pore (Esau & Cheadle, 1959), leading to a misapprehension of the cross‐sectional area of end walls available for phloem sap flow, especially of the smaller pores of compound end walls.

The phylogenetic distribution of SE end wall types presented here demonstrates that both characteristics evolved independently on numerous occasions, that is, the convergent evolution of this trait. The occurrence of plants with compound end walls in families whose precursor featured simple end walls contradicts the hypothesis of a unidirectional evolutionary trend towards simple end walls (Zahur, 1959; Esau, 1969; Chavan, 1986). Our observation is supported by paleobotanical analyses showing that some of the first angiosperms from the Carboniferous featured transverse SE end walls in lineages that afterwards exhibited compound end walls (Taylor, 1990). The trend towards simple end walls was postulated in connection with the assumption that they provide less resistance than compound end walls, which has been shown to be wrong here. Indeed, our data indicate that hydraulic resistance is not decisive in the evolution of end wall types. Height‐related scaling of SE resistance as well as average end wall resistance is similar for woody angiosperms with simple or compound end walls. In addition, the comparison of closely related species provided no indication of hydraulic resistance influencing the evolution of end wall type.

### Which factors drive evolution towards a specific end wall type?

If not hydraulic resistance, what could be the driver for the evolution of different end wall anatomies? Factors indirectly linked to phloem transport, such as leaf length as an indicator for phloem loading capacity and growth rates as an indicator for sink strength, showed no correlation with end wall type and can therefore be excluded as evolutionary driver. It has been proposed that a change from compound to simple end walls parallels the trends for tracheary elements in the xylem (Bailey, 1953; Carlquist, 1975). However, studies that characterized the bark and wood structure of many species of a specific family did not support this hypothesis (Donghua & Xinzeng, 1993; Kotina & Oskolski, 2010; Pace *et al*., 2015). Mullendore *et al*. (2010), upon not finding a correlation of sieve pore size with transport velocity in herbaceous angiosperms, suggested that the ability for plugging the pores could be a critical factor in a plant's reaction to stress. However, the variation in end wall anatomy, including sieve pore radius, of closely related species does not seem to support an evolutionary connection between SE end wall anatomy and efficiency of the wound response in woody plants.

A change in climate was recently identified as a trigger for the evolutionary shift from simple to scalariform xylem vessel perforation plates in the Adoxaceae (Lens *et al*., 2016). Phloem SE end wall type, however, was found not to correlate with the plant's native climate zone in the woody angiosperms investigated here. However, by using only broad categories, our analysis might have missed a connection between SE morphology and specific environmental variables. Water availability, in particular, which is a critical factor for phloem transport (Sevanto *et al*., 2011), could potentially drive adaptations in phloem anatomy. In the eudicot *Styrax*, SEs bear simple end walls in the roots and compound end walls in the stems of species growing in a strong seasonally dry environment, but the end walls are always exclusively compound in species growing in more mesic habitats (Machado *et al*., 2005), suggesting a potential functional role of different end wall morphologies. More studies are needed to explore this connection between SE anatomy and environment.

Previous large‐scale studies of SE structure and its relation to other bark characteristics did not find significant correlations that could indicate coevolution, except between end wall type and mechanical tissue type as well as SE length (Zahur, 1959; Den Outer, 1983). As a connection between mechanical tissue and SE stability was indicated in the Bignonieae (Pace *et al*., 2015), it could be hypothesized that the collapse of SEs seen in species with scarce fibers is related to the typically co‐occurring simple end walls. However, an influence of end wall type on the rigidity of SEs appears unlikely in light of the relatively short length of end walls in comparison to the length of the SE. Indeed, our data, especially the comparison of species with different end wall types within the same genus, did not support the notion of coevolution with mechanical tissue. In contrast to SE end wall type, the mechanical tissue type appeared evolutionary‐conserved. It should be noted, however, that we analyzed only the mechanical tissue type and not mechanical tissue abundance, which was implicated in SE stability in the Bignonieae (Pace *et al*., 2015).

Our data are in agreement with all previous publications on the connection between SE end wall type and SE length (Zahur, 1959; Esau, 1969; Den Outer, 1983; Donghua & Xinzeng, 1993) in showing that the average SE length of woody angiosperm species with simple end walls is significantly lower than that of species with compound end walls. As, in species with compound end walls, the number of sieve areas scaled with SE length and both were shown to scale with plant height, it can be speculated that end wall type depends on SE length. As longer SEs mean fewer end walls along the transport path, it is an efficient way to reduce hydraulic resistance per unit length. Indeed, according to our data, the average number of end walls along 1 m of stem is 635 (SD ± 375) for gymnosperms, 2715 (SD ± 1042) for woody angiosperms with compound end walls and 4296 (SD ± 1597) for woody angiosperms with simple end walls. At the same time, the ratio of SE radius to length is *c*. 20% higher for woody angiosperms with simple end walls than for those with compound end walls. With regard to evolution, it could be hypothesized that a demand for increasing phloem conductivity, for example as a result of increasing height or decreased water availability (Ryan & Asao, 2014), drives the increase of either SE length or SE diameter. The end wall type would then be a consequence of which of the two strategies is followed. To test this hypothesis, a careful analysis of the developmental constraints with regard to SE shape has to be conducted. At the same time, a continued focus on SE physiology and a stronger combination of ecological and physiological approaches, as has been proposed recently (Savage *et al*., 2016), will be required to solve the newly opened question of what factor(s) drives phloem evolution.

## Author contributions

J.L. conceived the project, assembled the dataset, conducted the correlation analysis, calculated hydraulic resistances and contributed to assembling the phylogenetic trees. M.R.P. collected part of the samples, made anatomical measurements and assembled the phylogenetic trees. Y.L. collected part of the samples. Q.X. and S.C. contributed to data analysis. The manuscript was written by J.L. with participation from all authors.

## Supporting information

Please note: Wiley Blackwell are not responsible for the content or functionality of any Supporting Information supplied by the authors. Any queries (other than missing material) should be directed to the *New Phytologist* Central Office.

**Fig. S1** Phylogenetic distribution of the simple and compound type of sieve element end walls in families where both types occur.

**Table S1** Morphological and physiological data of the 447 species of woody plant used in this study.

**Table S2** Intraspecies variation of sieve element characteristics.
