# Supplementary material for: Height‐related scaling of phloem anatomy and the evolution of sieve element end wall types in woody plants
Source: New Phytol. 2016 Dec 9;214(1):245–56. doi: 10.1111/nph.14360 (PMC5347917; doi:10.1111/nph.14360)
Supplement: Supplementary file 1 — Fig. S1 Phylogenetic distribution of the simple and compound type of sieve element end walls in families where both types occur. [file NPH-214-245-s001.pdf]

Supporting information Fig. S1: Phylogenetic distribution of the simple and compound type of sieve element end walls in families where both types occur. White bars indicate simple end walls, black bars indicate compound end walls and grey bars indicate the occurrence of both types. Phylogenies based on Apocynaceae: Endress & Bruyns (2000), Araliaceae: Lowry *et al*. (2004), Celastraceae: Wenqing & Xinzeng (1993), Euphorbiaceae: Tokuoka (2007), Fabaceae: Sirichamorn et al. (2012), Brown et al. (2008), Fagaceae: Manos et al. (2001), Lamiaceae: Scheen et al. (2010), Malpighiaceae: Davis et al. 2001, Malvaceae: Bayer et al. (1999), Myrtaceae: Wilson et al. (2005), Rosaceae: Potter et al. (2007), Rubiaceae: Bremer (2009), Salicaceae: The UniProt Consortium (2015), Sapindaceae: Harrington (2005), Vitaceae: Jansen et al. (2006). The corresponding tree for Bignoniaceae can be found in Pace *et al*. (2015).

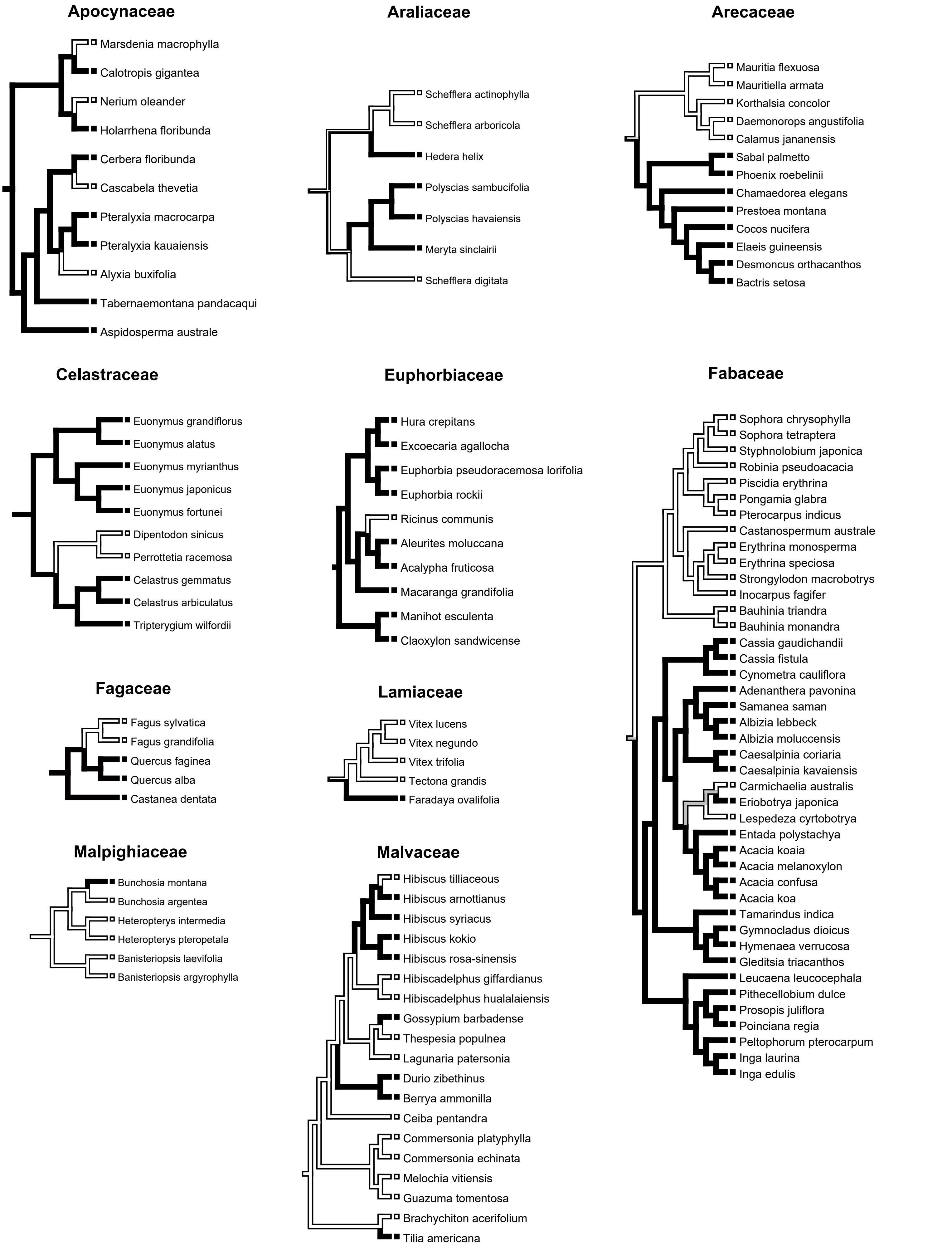

Supporting information Fig. S1 - Page 2

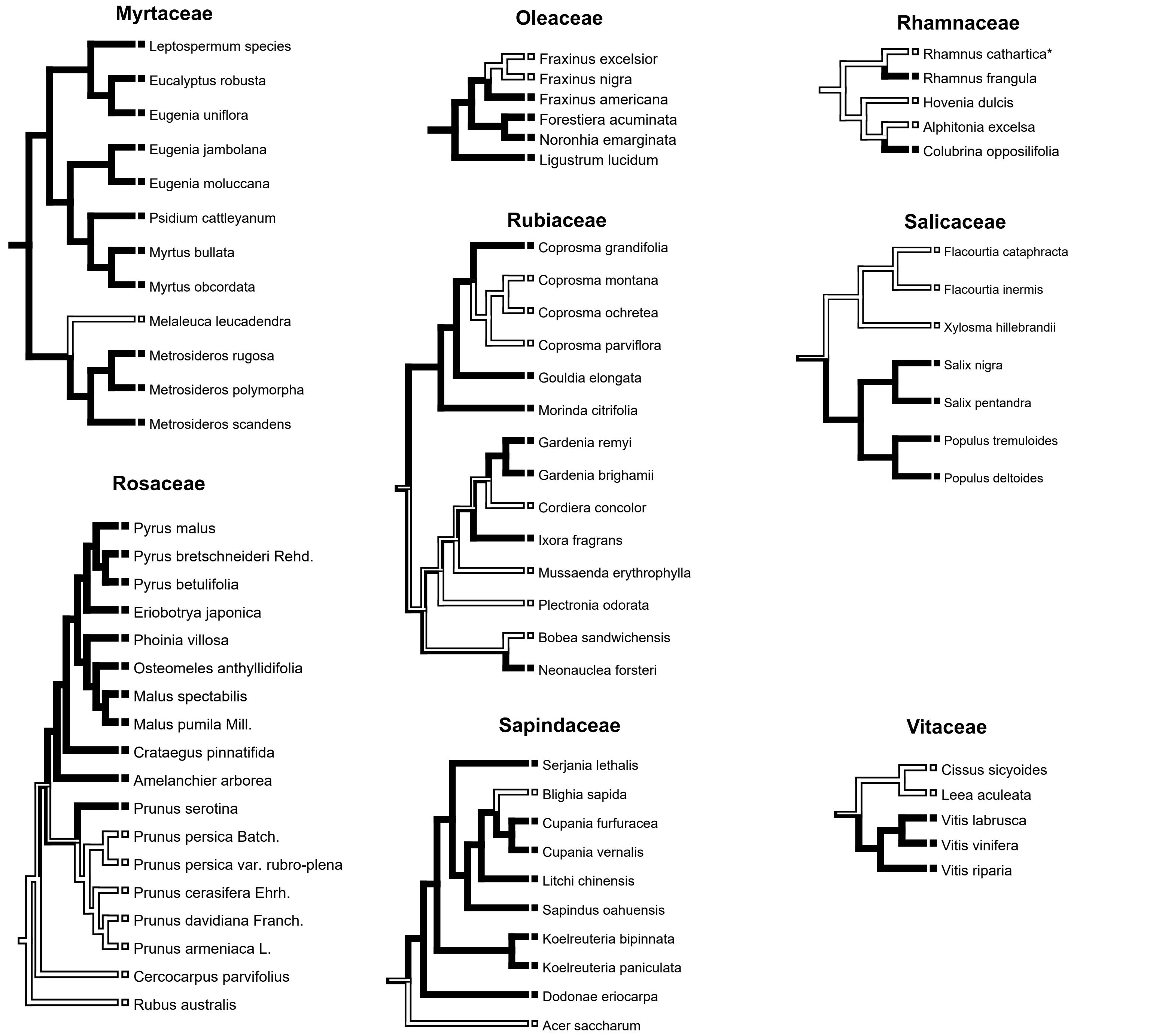

References

Bayer C, Fay MF, De Bruijn AY, Savolainen V, Morton CM, Kubitzki K, Alverson WS, Chase MW. 1999. Support for an expanded family concept of Malvaceae within a recircumscribed order Malvales: a combines analysis of plastid *atp*B and *rbc*L DNA sequences. *Botanical Journal of the Linnean Society* **129**: 267-303.

Bremer B. 2009. A review of molecular phylogenetic studies on Rubiaceae. *Annals of the Missouri Botanical Garden* **96**: 4-26.

Brown GK, Murphy DJ, Miller JT, Ladiges PY. 2008. Acacia s.s. and its relationship among tropical legumes, tribe Ingeae (Leguminosae: Mimosidae). *Systematic Botany* **33**: 739-751.

Davis CC, Anderson WR, Donoghue MJ. 2001. Phylogeny of Malpighiaceae: Evidence from chloroplast NDHF and TRNL-F nucleotide sequences. *American Journal of Botany* **88**: 1830-1846.

Endress ME, Bruyns PV. 2000. A revised classification of the Apocynaceae s.l. *The Botanical Review***66**: 1-56.

Harrington MG, Edwards KJ, Johnson SA, Chase MW, Gadek PA. 2005. Phylogenetic Inference in Sapindaceae sensu lato Using Plastid *mat*K and *rbc*L DNA Sequences. *Systematic Botany* **30**: 366-382.

Jansen RK, Kaittanis C, Lee SB, SaskiC, Tomkins J, Alverson AJ, Daniell H. 2006. Phylogenetic analyses of *Vitis*(Vitaceae) based on complete chloroplast genome sequences: effects of taxon sampling and phylogenetic methods on resolving relationships among rosids. *BMC Evolutionary Biology* **6**: 32.

Lowry PP, Plunkett GM, Wen J. 2004. Generic relationships in Araliaceae: looking into the crystal ball. *South African Journal of Botany* **70**: 382-392.

Manos PS, Zhou ZK, Cannon CH. 2001. Phylogenetic tests of reproductive trait evolution. *International Journal of Plant Sciences* **162**: 1361-1379.

Pace MR, Alcantara S, Lohmann LG, Angyalossy V. 2015. Secondary phloem diversity and evolution in Bignonieae (Bignoniaceae). *Annals of Botany* **116**: 333-358.

Potter D, Eriksson T, Evans RC, Oh S, Smedmark JE, Morgan DR, Kerr M, Robertson KR, Arsenault M, Dickinson TA, Campbell CS. 2007. Phylogeny and classification of Rosaceae. *Plant systematics and evolution***266**: 5–43.

Scheen AC, Bendiksby M Ryding O, Mathiesen C, Albert VA, Lindqvist C. 2010. Molecular phylogenetics, character evolution, and suprageneric classification of Lamioideae (Lamiaceae). *Annals of the Missouri Botanical Garden* **97**: 191-217.

Sirichamorn Y, Adema FB, Gravendeel B, van Welzen PC. 2012. Phylogeny of palaeotropic Derris-like taxa (Fabaceae) based on chloroplast and nuclear DNA sequences shows reorganization of (infra)generic classifications is needed. *American Journal of Botany* **99**: 1793-1808.

The UniProt Consortium. 2015. UniProt: a hub for protein information. *Nucleic Acids Research* **43**: 204-212.

TokuokaT. 2007. Molecular phylogenetic analysis of Euphorbiaceae sensu stricto based on plastid and nuclear DNA sequences and ovule and seed character evolution. *Journal of Plant Research* **120**: 511-522.

Wenqing Q, Xinzeng G. 1994. Comparative anatomy of secondary phloem in Celastraceae. *Acta Botanica Sinica* **36**: 779-784.

Wilson PG, O'Brien MM, Heslewood MM, Quinn CJ. 2005. Relationship within Myrtaceae sensu lato based on matK phylogeny. *Plant systematics and evolution* **251**: 3-19.
